# Buruli Ulcer Prevalence and Altitude, Benin

**DOI:** 10.3201/eid1701.100644

**Published:** 2011-01

**Authors:** Ghislain Emmanuel Sopoh, Roch Christian Johnson, Séverin Yehouénou Anagonou, Yves Thierry Barogui, Ange Dodji Dossou, Jean Gabin Houézo, Delphin Mavingha Phanzu, Brice Hughes Tente, Wayne M. Meyers, Françoise Portaels

**Affiliations:** Author affiliations: Centre de Dépistage et de Traitement de l’Ulcère de Buruli d’Allada, Allada, Bénin (G.E. Sopoh, A.D. Dossou, J.G. Houezo);; Programme National de Lutte Contre la Lèpre et l’Ulcère de Buruli, Cotonou, Bénin (R.C. Johnson);; Laboratoire de Référence des Mycobactéries, Cotonou (S.Y. Anagonou);; Centre de Dépistage et de Traitement de l’Ulcère de Buruli de Lalo, Lalo, Bénin (Y.T. Barogui);; Institut Médical Evangélique, Kimpese, Bas-Congo, Democratic Republic of Congo (D.M. Phanzu);; Université d'Abomey-Calavi, Bénin (B.H. Tente);; Armed Forces Institute of Pathology, Washington, DC, USA (W.M. Meyers);; Institute of Tropical Medicine, Antwerp, Belgium (F. Portaels)

**Keywords:** Buruli ulcer, bacteria, prevalence, altitude, distribution, environment, risk factor, Benin, letter

**To the Editor:** Buruli ulcer (BU), caused by *Mycobacterium ulcerans*, is one of 13 recently classified neglected tropical diseases (*1*). Little is known about factors influencing its focal distribution. In Benin, altitude may play a role in such distribution of BU.

Incidence, prevalence, and other health-related data are usually reported at national or district levels. These data convey the importance of the disease but do not show the wide variations existing at the village level. Data from the surveillance system (*2*) and surveys (*3**–**6*) in Benin have shown that BU-endemic areas are confined to the southern regions. Substantial variability in endemicity levels have been detected from 1 department to another, at the district and village levels, and from year to year (*2**–**5*).

However, some districts (Lalo in the Mono-Couffo Department, Ouinhi in the Zou Department; Zê in the Atlantique Department; and Adjohoun, Bonou, and Dangbo in the Oueme Department) remain the most persistently BU-endemic from year to year. In addition, these BU-endemic districts are all located at the same latitude. A map of these districts can be superimposed on the Lama depression (a median band, oriented from west to east, that forms a large area at a low elevation, 130 km long with a width from 5 km in the area of Tchi in Lalo to 25 km in the area of Issaba in Pobê) (*7*). This factor prompted us to investigate whether variations in altitude correlate with BU prevalence.

Using a Garmin eTrex global positioning system (Olathe, KS, USA), we collected precise geographic coordinates, including altitude, for each village in 2 persistently BU-endemic districts of the Atlantique Department. We chose districts where BU endemicity was high (Zê) and low (Toffo) (prevalences 52.0 and 7.8/10,000 inhabitants, respectively) (*3**,**5*). On the basis of routine data collected during 2005–2009, we calculated the prevalence of BU in each village of these districts and correlated it with the altitude of the village, first by mapping with Healthmapper 4.3.2 (http://healthmapper.software.informer.com) and then with statistical analyses by using Epi Info 3.5.1 (Centers for Disease Control and Prevention, Atlanta. GA, USA).

We found that highly BU-endemic villages are located most often in lowland areas (Figure A1). The mean prevalence of BU was 60.7/10,000 inhabitants in villages with elevations <50 m, which was significantly higher than the prevalence in villages with elevations 50–100 meters (10.2/10,000 inhabitants) and that of villages with elevations >100 meters (5.4/10,000 inhabitants) (p = 0.0003; Kruskal-Wallis test).

In addition, we performed a simple linear regression, including all villages (model A) and only BU-endemic villages (prevalence ≠ 0) (model B). Model A showed that at 0 altitude, the expected prevalence of BU was 26.7/10,000 inhabitants. This prevalence decreased by 0.1/10,000 inhabitants for each meter of increase in altitude (correlation coefficient 0.20; coefficient of determination 4%). Model B demonstrated that at 0 altitude, the expected prevalence was 89.6/10,000 inhabitants. This prevalence decreased by 0.7/10,000 inhabitants for each meter of increase in altitude (correlation coefficient 0.50; coefficient of determination 25%). Therefore, we conclude that a low but significant linear relationship exists between altitude and BU prevalence in disease-endemic villages. Thus, altitude may be 1 factor in determining variations in prevalence (4% for all villages and 25% for BU-endemic villages).

The focal distribution of BU was discussed in 1974 by Meyers et al. in Zaire (*8*). In the Bas-Congo Province, although the concentration of BU in Songololo was high, the nearby broad Bangu plateau, ≈300 meters higher than Songololo, was devoid of BU (D.M. Phanzu, unpub. data). Soil and geologic features (e.g., chemical composition of substrata; vegetation, fauna, and pH of swamps) were raised as environmental factors that might explain this focal distribution (*8**,**9*). The focal distribution of BU was also described by Johnson et al., who found an inverse relationship between the prevalence of the disease in Lalo District villages and distance from the Couffo River (*4*).

Few studies have investigated environmental risk factors (other than water-related) possibly related to the prevalence of BU. In 2008, Wagner et al. suggested that villages with higher prevalence rates were located in areas of low elevation. They associated the high prevalence of BU with farming activities that occurred primarily at low elevations (*10*). Our results are similar, but we have provided additional quantification of the relationship between prevalence and altitude.

One reasonable explanation for the relationship between altitude and BU prevalence is that because lowlands tend to be wetter than higher grounds, they provide more favorable conditions for the proliferation and spread of the etiologic agent. Furthermore, persons are more apt to frequent these wetter lowlands to plant and tend their crops, thus becoming vulnerable to infectious agents in the area.

An extension of this study to all BU-endemic villages is needed to further refine our results. The endemicity of BU is multifactorial; however, our results suggest that altitude should be included in future analytical models of environmental risk factors for this disease.

## Figures and Tables

**Figure A1:**
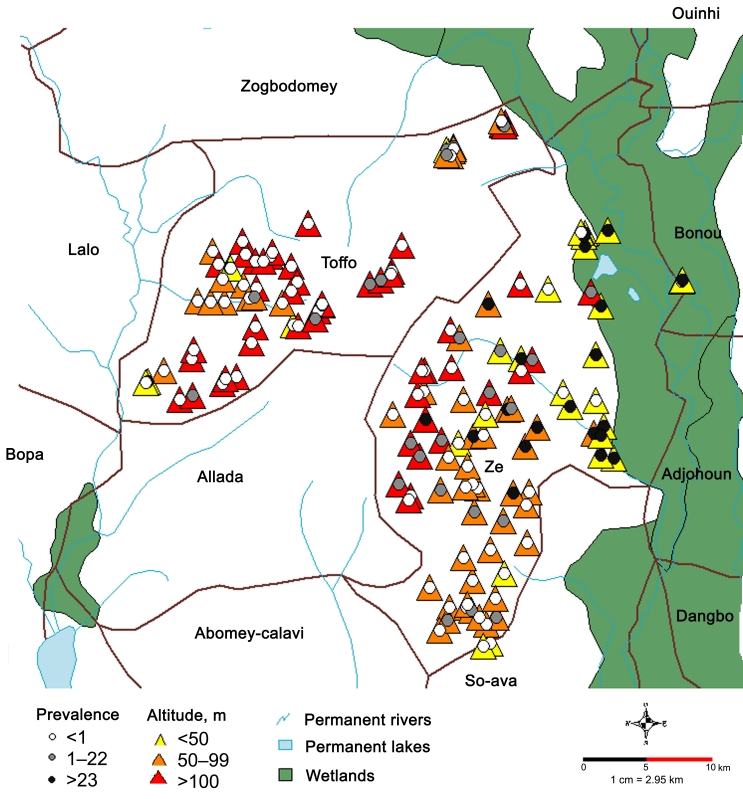
Correlation between altitude and prevalence of Buruli ulcer for villages of the districts of Zê and Toffo in the Atlantique Department of Benin, 2005–2009.
